# Utility of tumor and non-tumor biopsies during percutaneous radiofrequency ablation for hepatocellular carcinoma

**DOI:** 10.1016/j.jhepr.2025.101430

**Published:** 2025-04-22

**Authors:** Lorraine Blaise, Marianne Ziol, Claudia Campani, Nathalie Ganne-Carrie, Pierre Nahon, Gisele Nkontchou, Jessica Zucman-Rossi, Lucie Del Pozo, Nathalie Barget, Carina Boros, Elvire Desjonqueres, Alix Demory, Veronique Grando, Lorenzo Pescatori, Olivier Seror, Olivier Sutter, Jean-Charles Nault

**Affiliations:** 1 AP-HP, Hôpital Avicenne, Service d’Hepatologie, Bobigny, 93000, France; 2 AP-HP, Hôpital Avicenne, Service d’Anatomopathologie, Bobigny, 93000, France; 3 Unité de Formation et de Recherche Santé Médecine et Biologie Humaine, Université Sorbonne Paris Nord, Bobigny, 93000, France; 4 Centre de Recherche des Cordeliers, Sorbonne Université, Inserm, Université de Paris, Team ‘Functional Genomics of Solid Tumors’, F-75006 Paris, France; 5 Department of Experimental and Clinical Medicine, INternal Medicine and Hepatology Unit, University of Firenze, Florence, 50100, Italy; 6 Plateforme de Ressource Biologique HUPSSD, BB-0033-00027, AP-HP, Hôpital Avicenne, Bobigny, 93000, France; 7 AP-HP, Hôpital Avicenne, Service de Radiologie Interventionnelle, Bobigny, 93000, France

**Keywords:** Biopsy, Hepatocellular carcinoma, Diagnosis, Prognosis, Molecular biology

## Abstract

**Background & Aims:**

We aimed to assess the safety and diagnostic/prognostic value of tumor and non-tumor biopsies systematically collected during radiofrequency ablation (RFA) of hepatocellular carcinoma (HCC).

**Methods:**

We prospectively included patients with a first diagnosis of HCC who underwent tumor and non-tumor biopsies during percutaneous RFA between 2015 and 2021. We analyzed the complications, percentage of diagnostic tumor biopsies, ability to perform molecular biology, and the non-tumor liver biopsy results, and correlated histology with prior non-invasive diagnosis and oncological outcomes.

**Results:**

In total, 248 patients (86% male, median age 68) with 302 tumors treated by RFA and with available tumor biopsy were included. HCC was single in 78% and bifocal in 21% of patients, with a median size of 24 mm. Bleeding occurred in six cases (1.9%) without related deaths. Biopsies enabled HCC diagnosis in 66% of cases, with positivity linked to nodule size (*p* <0.0001), location (*p* = 0.04), and ultrasound visibility (*p* = 0.004). A discrepancy between prior tumor board and subsequent histological diagnosis was observed in 5% of cases. Among the 302 biopsies, 34% were non-diagnostic, 61% were HCC, 3% were cholangiocarcinoma (CCA)/hepatocholangiocarcinoma (cHCC-CCA), and 2% were dysplastic nodules. Survival was shorter in patients with CCA/cHCC-CCA (*p* <0.001). Macrotrabecular-massive HCC was associated with higher rates of global tumor recurrence (*p* = 0.037). More than 25% of tumor cells in paraffin-embedded samples were associated with expression of cancer genes on transcriptomic analysis of the corresponding frozen samples, assuring their usefulness for molecular analysis. In non-tumor biopsies, cirrhosis was histologically confirmed in 82% of cases, with a 15% discrepancy between diagnosis of cirrhosis at tumor board and on biopsy.

**Conclusion:**

Systematic tumor and non-tumor biopsy during RFA for a first diagnosis of HCC is feasible, safe, and brings valuable diagnostic, therapeutic, and prognostic data.

**Impact and implications:**

Tumor and non-tumor biopsies during radiofrequency ablation for hepatocellular carcinoma are both safe and feasible, with a low complication rate and no procedure-related deaths. We demonstrated the diagnostic utility of biopsies, revealing discrepancies in 5% of cases between tumor board and histological results, which could refine patient management strategies. We identified significant associations between hepatocellular carcinoma subtypes and recurrence rates, and found that molecular analysis based on frozen tumor samples is feasible in most cases and could be guided by the percentage of tumors cells on paraffin-embedded samples.

## Highlights

• Systematic tumor and non-tumor biopsies during RFA of HCC are safe.
• Tumor biopsy confirmed HCC in 66% of cases and revealed a 5% discrepancy (mostly CCA /cHCC-CCA).
• Non-tumor biopsy confirmed cirrhosis in 82% of cases, with a 15% discrepancy from MTB diagnosis.

## Graphical abstract

**Figure:**
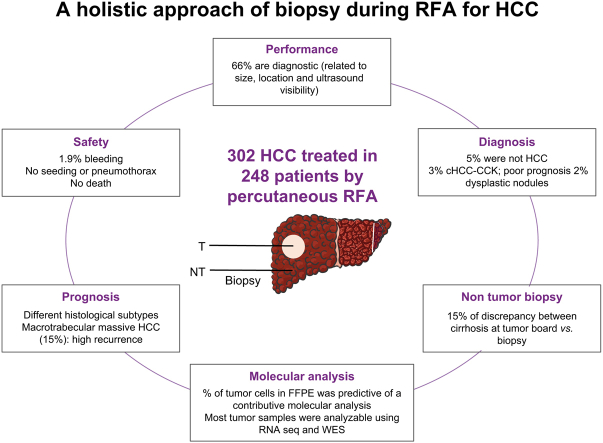

## Introduction

The implementation of imaging for non-invasive diagnosis of hepatocellular carcinoma (HCC) began during the early 2000s and represents one of the unique examples in oncology where histology is not always required for diagnosis.^1^^,^^2^ In 2024, the Liver Imaging Reporting and Data System (LI-RADS) was the most widely used system to assess the probability of HCC in cases of liver lesions developed on chronic liver disease.^3^^,^^4^ This classification is often used during multidisciplinary tumor board (MTB) meetings to guide discussions on whether a tumor biopsy is necessary or if a fully non-invasive diagnosis of HCC is feasible. Currently, tumor biopsies are mainly performed for nodules that develop in non-cirrhotic livers or for atypical nodules detected/found on imaging in patients with cirrhosis.^1^^,^^2^ Furthermore, the ability to perform non-invasive HCC diagnosis was recently extended to patients with chronic HBV without cirrhosis, as a result of the high pretest probability and good sensitivity and specificity of non-invasive criteria in this clinical setting.^5^

Several reasons justify the use of non-invasive criteria. First, tumor biopsies carry risks, such as bleeding or tumor seeding, even though these complications are rare, with a bleeding rate of ∼1–2% in ultrasound-guided percutaneous biopsy.[6], [7], [8] Moreover, the diagnostic yield of biopsies is moderate for nodules 1–2 cm in size, which is the target size for HCC screening in patients with cirrhosis, with only 60–70% of biopsies providing positive results.[6], [7], [8] Lastly, biopsy currently has no theranostic value in clinical practice, because the identification of actionable mutations to guide targeted therapies has not yet been validated.^9^

However, the use of non-invasive imaging for HCC diagnosis has led to several implications in the field. Obtaining tumor samples could improve our understanding of the natural history of liver cancer and its genomic landscape, potentially leading to the identification of new prognostic biomarkers useful for stratifying adjuvant treatments in future clinical trials.^10^ Furthermore, in most advanced HCC clinical trials, histological diagnosis is often unavailable, limiting the use of tumor biopsies in translational research aimed at identifying biomarkers predictive of immunotherapy response.^11^ In this context, a comprehensive evaluation of the role of systematic biopsies in patients with HCC is needed to assess the safety of the procedure, its impact on clinical diagnosis and prognosis, and its potential future role in molecularly guided therapy.^12^ To address this, performing both tumor and non-tumor biopsies during percutaneous radiofrequency ablation (RFA) for the initial diagnosis of HCC could be a valuable approach.^13^ This would enable reassessment of the role of systematic tumor biopsy in patients treated with percutaneous ablation who often lack histological confirmation of their cancer, unlike those undergoing resection or transplantation who undergo a systematic histological diagnosis.

Therefore, our primary aim here was to prospectively evaluate the safety, diagnostic yield, prognostic impact, and potential uses for theranostic of systematic non-tumor and tumor biopsies performed during initial RFA treatment for HCC.

## Patients and methods

### Inclusion criteria

We prospectively included all patients referred and treated at a tertiary center (Avicenne Hospital, France) between January 1, 2015 and December 31, 2021 who met the following inclusion criteria: (1) diagnosis of HCC using non-invasive criteria at the MTB; (2) treatment by a first session of RFA in a curative intent; (3) no previous HCC treatments; Barcelona Clinic Liver Cancer (BCLC) stage 0 or A; (4) aged over 18 years; and (5) systematic tumor and non-tumor biopsy performed during the procedure.

In France, national guidelines for the management of patients with HCC recognize histology as the gold standard for the diagnosis of HCC, while non-invasive diagnosis is considered a diagnostic alternative in cases of cirrhosis.^1^ Following this guideline, we systematically and prospectively performed in our unit both tumor and non-tumor biopsies during the first RFA for HCC to enable histological analysis. Exceptions were made only in the following cases: (1) when a biopsy had already been performed before the ablation and confirmed HCC; or (2) when the tumor biopsy was considered technically too challenging by our radiologists. All the patients gave written consent to analyze the residual tumor samples not used for diagnosis, for a research purpose (MUTHEC NCT03071458, CCPPRB Paris Saint-Louis IRB00003835 extended in ISP/PRC/CP/2019-025).

The diagnosis and treatment of each patient were discussed at MTB comprising hepatogastroenterologists, oncologists, liver surgeons and transplant specialists, interventional radiologists, and pathologists. We recorded the conclusion of the MTB regarding the stage of fibrosis of the non-tumor liver and distinguished patients with and without cirrhosis (based on data available at the time of the MTB, including previous non-tumor liver biopsy, transient elastography, and a combination of clinical, biological, and imaging data) to compare these data with the histological stage of fibrosis observed in the per-ablation non-tumor biopsy. We also recorded the median value of transient elastography if performed within the 6 months preceding the ablation and if the exam met the quality control standards (at least 10 valid individual measurements with IQR/median ratio <30%) using the M probe for patients with a BMI <30 kg/m^2^ and the XL probe if the BMI was >30 kg/m^2^. We also collected clinical (age, sex, etiologies of the underlying liver disease, and uses of anticoagulation and antiplatelet agent) and biological data (Child-Pugh Score, platelet count, serum alpha-fetoprotein [AFP] level, prothrombin time, and activated partial thromboplastin time) immediately before the percutaneous RFA.

### Imaging analysis

All patients underwent either contrast-enhanced liver magnetic resonance imaging (MRI) with lung computerized tomography (CT) (152 patients) or triple-phase contrast-enhanced thoraco-abdominal CT (128 patients) before ablation to perform diagnosis and tumor staging. We recorded the number and size of tumors and staged them according to the BCLC classification.^14^ Imaging was reviewed (available for 284 nodules among the 302 nodules), by one radiologist (OSu) to classify the lesion according to the LI-RADS 2018.^3^

### Percutaneous radiofrequency ablation

All procedures were performed under general anesthesia. Platelet transfusion was administered 1 h before the percutaneous RFA in patients with a platelet count <50,000/mm^3^. We collected data on the type of RFA used, distinguishing between monopolar and multibipolar techniques, the number of sessions required to achieve complete ablation, and whether the tumor was visible in ultrasonography alone. All monopolar RFAs (n = 6) were performed with intratumoral placement of the probe, whereas all multibipolar RFAs (n = 241) were performed following the no-touch concept.^15^^,^^16^ A median of three electrodes (IQR: 3–4) was used per patient, with a median energy delivery of 111 kJ (IQR: 61–187) and a median ablation duration of 26 min (IQR: 18–41). The track ablations were systematically performed during needle(s) withdrawal. Complete ablation was confirmed when no residual enhancement adjacent to the ablation zone was observed during the arterial phase on imaging (CT or MRI) performed 1 month after RFA. If ablation was incomplete, up to two additional RFA ablative procedures were performed. Treatment failure was defined as the inability to achieve complete ablation after three procedures, and these patients were considered to have local tumor progression in statistical analyses. Adverse events related to the treatment were documented throughout all ablation sessions according to the Dindo-Clavien Classification.^17^ We also specifically assessed the following adverse events as potentially related to tumor biopsy: bleeding, pneumothorax, and tumor seeding.

Patients underwent follow-up evaluations, including clinical, biological, and imaging assessments with triple-phase contrast-enhanced CT or MRI. Follow-up was initially conducted 1 month post ablation, then every 3 months during the first 2 years, and every 6 months thereafter. Follow-up continued until death or the last recorded visit on June 1, 2024.

### Realization of tumor and non-tumor biopsy per procedure

At the beginning of the RFA procedure, once the patient was under general anesthesia, prior contrast-enhanced 3D liver imaging (preoperative CT/MRI) or Cone Beam CT reconstruction acquired at the start of the procedure was synchronized with ultrasound using the image-fusion module of the ultrasound machine (Logic E9, GE Healthcare, Chicago, IL, USA). This image-fusion method was systematically used and enabled the ablation of tumors that were inconspicuous or poorly visible under ultrasound alone. A first non-tumor biopsy was performed along the path to the tumor, followed by two tumor biopsies (one to be formalin fixed and one to be conserved immediately in nitrogen at -80 °C for subsequent molecular analysis) targeting either the visible nodule or its presumed location if it remained undetectable despite image fusion. An automatic full-core 18-gauge biopsy system (BioPince, Argon Medical, Athens, TX, USA) was used with its dedicated 17-gauge cannula, and the biopsy length was set to 13, 23, or 33 mm depending on the tumor size. To minimize the risk of bleeding, the coaxial cannula was left in place after the biopsies. Once the thermal energy had been delivered, the biopsy cannula was progressively removed simultaneously with the closest ablative needle during the tract ablation.

### DNA and RNA extraction, quantification, and qualification

We collected a total of 241 frozen liver tumor biopsies during tumor ablation from patients treated for HCC: 185 HCC from this series of patients and 56 HCC collected during other types of percutaneous ablation. All tumors had a histologically proven HCC in the formalin-fixed paraffin-embedded (FFPE) samples with various percentages of tumor cells that pathologists classified as <25%, 25–50%, 50–75%, and >75%. We also analyzed 148 frozen samples of non-tumor liver (including 108 cirrhotic and 40 non-cirrhotic liver) from 146 liver biopsies and two surgical samples already available in the laboratory. Genomic DNA from frozen surgical samples was extracted using the Maxwell® RSC Tissue DNA Kit (#AS1610, Promega, Madison, WI, USA), and RNA from frozen surgical samples was extracted using the Maxwell® RSC simplyRNA Tissue Kit (#AS1340, Promega). DNA and RNA from frozen biopsies were extracted using the AllPrep DNA/RNA/miRNA Universal Kit (#80224, QIAGEN, Venlo, The Netherlands) according to the manufacturer’s instructions. DNA and RNA were quantified using the Qubit™ dsDNA BR Assay Kit (cat# Q32850, Life Technologies – Thermo Fisher Scientific, Waltham, MA, USA) and a NanoDrop spectrophotometer at 260 nm respectively, while quality was assessed by agarose gel electrophoresis. A concentration of 15 ng/μl in a final volume of 60 μl was considered as sufficient to perform RNA sequencing (RNAseq) and whole-exome sequencing (WES) if the quality was adequate.

### Quantitative RT-PCR and transcriptomic analysis

We assessed mRNA expression by quantitative RT-PCR performing reverse transcription of 500 ng of RNA using the High-Capacity Transcription Kit (#4368813, Life Technologies) and assessing gene expression using TaqMan predesigned probes (Life Technologies) on Fluidigm 96 dynamic arrays with the Bio-Mark Real-Time PCR system, as previously described.^18^ A panel of 36 genes was selected on their ability to differentiate HCC from non-tumor liver tissues (Table S1). Expression level (Ct values) was assessed using Fluidigm Real-Time PCR Analysis software (4.1.3) and gene expression data were expressed using the 2DDCt method relative to ribosomal 18S (R18S) and the expression level of the corresponding gene in normal liver samples. Hierarchical clustering was performed on 241 HCC and 148 non-tumor liver samples, based on the normalized expression of the 36 genes. The hclust R function was used with Euclidean distance and Ward.D2 linkage method, after median-centering the data.

### Histological analysis of tumor and non-tumor liver samples

All tumor and non-tumor biopsies were reviewed by an expert pathologist (MZ). A diagnostic tumor biopsy was defined by the presence of tumor tissue (regardless of the tumor histology) at pathological reviewing, whereas a non-diagnostic tumor biopsy showed only non-tumor liver tissue. Moreover, the percentages of tumor cells were assessed on FFPE by the same pathologist. HCC samples were classified into different histological subtypes of HCC according to WHO Classification 2018: not otherwise specified (NOS-HCC), clear-cell HCC, steatohepatitic (SH-HCC), scirrhous HCC, lymphoepithelioma-like HCC, and macrotrabecular-massive (MTM-HCC) HCC.^19^ We also recorded the degree of differentiation of the tumor according to the WHO classification stage: well-differentiated HCC, moderately differentiated HCC, and poorly differentiated HCC. Finally, the degree of fibrosis of the non-tumor liver samples was classified from F0 to F4.

### Statistical analysis

Categorical variables were represented by number (percentages) and compared using either the Fisher test (two groups) or the Chi square test (more than two groups). Continuous variables were represented as median (IQR) and compared using the Mann-Whitney *U* test (two groups) or the Kruskall–Wallis test (more than two groups). Overall survival (OS) was defined as the time from the date of RFA to either the date of death or the most recent follow-up visit. Recurrence-free survival (RFS) was calculated from the date of RFA to the occurrence of tumor recurrence, death, or the most recent follow-up visit. Time to recurrence (TTR) was measured from the date of RFA to the occurrence of tumor recurrence or the most recent follow-up visit. Tumor recurrence was further categorized as either local tumor progression (recurrence adjacent to or within the ablation zone) or distant recurrence (recurrence in other segments or in the same segment but away from the treated area). Oncological outcomes (local recurrence, distant recurrence, overall tumor recurrence, RFS, and OS) were represented using the Kaplan–Meier methods with the log rank test. Variables associated with oncological outcomes were assessed using Cox univariate analysis, and, for variables with *p* <0.1, using Cox multivariate analysis. We performed competing risk analysis for tumor recurrence with death as a competitive event using the Fine and Gray method. All statistical analyses were performed using the R software; *p* <0.05 (two sided) was considered statistically significant.

## Results

### Description of the population and oncological outcomes

**Fig. 1:**
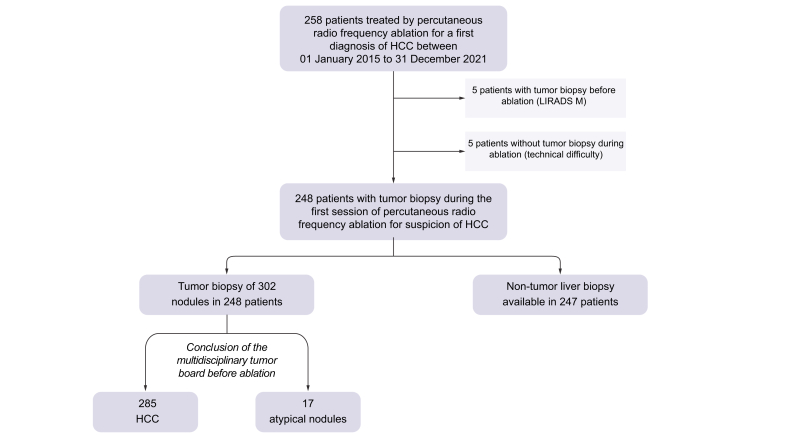
Study flow chart. HCC, hepatocellular carcinoma; LI-RADS, Liver Imaging Reporting and Data System.

A total of 248 patients with 302 nodules were treated by percutaneous RFA for a first diagnosis of HCC (Fig. 1). Among these 302 nodules, 285 were classified as typical HCC, while 17 were initially considered as atypical by the MTB. All atypical nodules (LIRADS 3) were associated with a typical HCC lesion in the same patient and the MTB decided to treat both lesions by percutaneous RFA in the same session. Most of the patients were male (86%) with a median age of 68 years old (IQR: 59–74) (Table 1). Of the patients, 87% had cirrhosis (based on the results of the non-tumor liver biopsy done per procedure). The causes of the underlying liver diseases were chronic HCV (23%), chronic HBV (15%), metabolic syndrome (42%), and/or chronic alcohol intake (54%). Furthermore, 78% of patients had a uninodular lesion of a median size of 24 mm (IQR: 19-31).

**Table 1:** Description of the 248 patients included in the study.

Variable	Available data	Whole population (N = 248 patients)
		n (%) or median (IQR)
Age (years)	248	68 (59–74)
Sex (male)	248	214 (86%)
BCLC 0	248	73 (30%)
BCLC A	248	175 (70%)
Size of main tumor (mm)	248	24 (19–31)
Size of main tumor >20 mm	248	155 (62.5%)
Size of main tumor >30 mm	248	63 (25.4%)
Uninodular lesion	248	193 (78%)
Fibrosis: non-tumor liver at MTB		
Non-cirrhotic	248	28 (11%)
Cirrhotic	248	220 (89%)
Fibrosis: non-tumor liver on per RFA biopsy		
F0–F1	247	18 (7.3%)
F2	247	13 (5.3%)
F3	247	13 (5.3%)
F4	247	203 (82%)
Chronic HCV	248	56 (23%)
Chronic HBV	248	38 (15%)
Metabolic syndrome	248	104 (42%)
Chronic alcohol intake	248	133 (54%)
Child-Pugh A	220	218 (90%)
Serum AFP level (ng/ml)	243	7 (4-27)
Platelet count (mm^3^)	239	132,000 (95,000–174,000)
Anticoagulation therapy	239	23 (0.6%)
Antiplatelet therapy	239	50 (21%)

Child-Pugh Score was calculated only in patients with cirrhosis defined by MTB.

AFP, alpha foetoprotein; BCLC, Barcelona Clinic Liver Cancer Classification; MTB, multidisciplinary tumor board; RFA, radiofrequency ablation.

**Table 2:** Description of the 302 tumors included in the study.

Variable	Available data	n (%) or median (IQR)
Tumor size (mm)	302	22 (17–30)
Tumor size >20 mm	302	136 (55%)
Tumor size >30 mm	302	66 (21.9%)
Tumor localization		
I	301	4 (1.3%)
II		23 (7.6%)
III		39 (13%)
IV		46 (15%)
V		37 (12%)
VI		50 (17%)
VII		49 (16%)
VIII		53 (18%)
LI-RADS classification		
3	284	12 (4%)
4		53 (19%)
5		219 (77%)
Detectable on ultrasonography	292	268 (92%)
Diagnosis at tumor biopsy		
HCC	302	184 (61%)
Hepatocholangiocarcinoma		6 (2 %)
Cholangiocarcinoma		3 (1.0%)
Dysplastic nodules		7 (2.3%)
Non-diagnostic biopsy		102 (34%)
**Description of histologically proven HCC**		
Not otherwise specified	176	96 (55%)
Steatohepatitic		34 (19%)
Scirrhous		11 (6.3%)
Lymphoepithelioma-like		4 (2.3%)
Macrotrabecular massive		26 (15%)
Clear cell		5 (2.8%)
Well-differentiated HCC	182	92 (51%)
Moderately differentiated HCC		60 (33%)
Poorly differentiated HCC		30 (16%)

HCC, hepatocellular carcinoma; LI-RADS, Liver Imaging Reporting and Data System.

A total of 24 patients (9.8%) experienced Grade III or more adverse events following ablations, including 11 Grade III (4.5%), 11 Grade IV (4.5%) and two Grade V (treatment-related death, 0.8%). Complete tumor ablation was achieved in 239 patients (97% of cases), after one session of RFA for 81%, after two sessions for 17%, and after three sessions for 2% of the patients. The median OS was 64.5 months, with 71% of patients alive at 3 years and 57% at 5 years; the median RFS was 18.3 months, with 30% of patients alive without recurrence at 3 years and 15% at 5 years. The median time to global recurrence was 22.5 months, with 64% of tumor recurrence at 3 years and 77% at 5 years. The rate of local tumor recurrence, including initial treatment failures, was 22% at 3 years and 25% at 5 years. The rate of distant recurrence was 56% at 3 years and 69% at 5 years.

### Safety and performance of tumor biopsy

Next, we analyzed the adverse events potentially related to tumor and non-tumor biopsy (bleeding, pneumothorax, and tumor seeding). We observed no cases of pneumothorax or tumor seeding in our patients. Six patients (incidence of 2.4% per patients and 1.9% per nodule treated) experienced post-RFA bleeding, including two hematomas of the skin in relation to the ablation tract, two liver hematomas, one hemothorax, and one hemoperitoneum. Only the two patients with hemothorax and hemoperitoneum required blood transfusions, with hemoperitoneum requiring emergency embolization and hemothorax necessitating drainage. The other four patients were managed conservatively, without any intervention or blood transfusion. No deaths occurred related to bleeding. Interestingly, 50 patients underwent biopsy and RFA while taking an antiplatelet agent (aspirin) and no significant difference in bleeding incidence (0%) was observed compared with patients not taking antiplatelet therapy (3%, *p* = 0.35). Moreover, 23 patients received RFA while taking curative anticoagulation. In contrast to antiplatelet agents, anticoagulation was stopped before treatment and reintroduced after ablation. The rate of bleeding was not significantly different between patients taking anticoagulant therapy compared with those not taking anticoagulant therapy (*p* = 0.11). Interestingly, we did not observe an increased risk of bleeding in the 76 patients with a platelet count <100,000/mm^3^ (5% increased risk of bleeding if <100,000/mm^3^ *vs.* 1% if >100,000/mm^3^, *p* = 0.08).

**Fig. 2:**
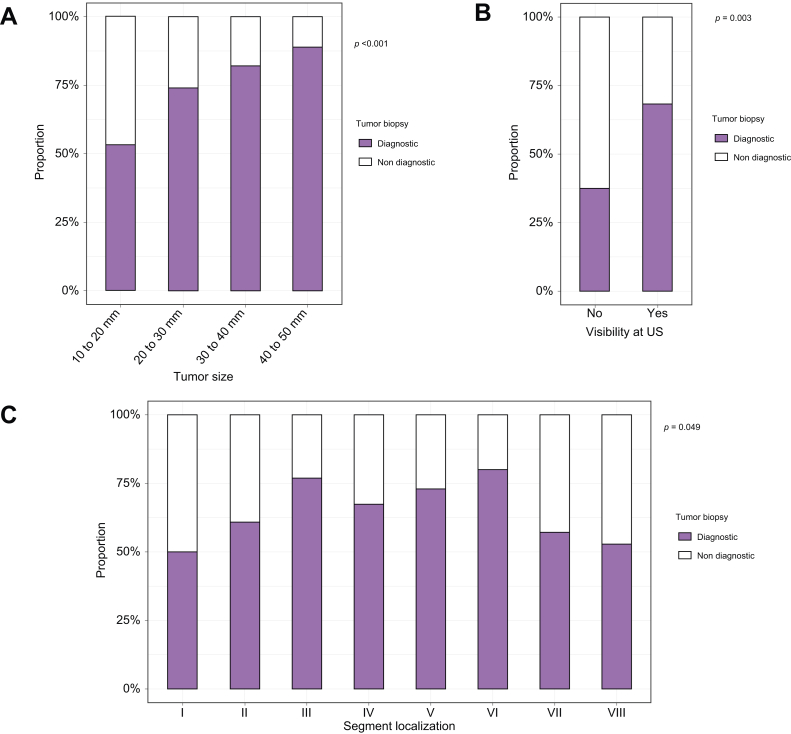
Variables associated with diagnostic tumor biopsy performed during radiofrequency ablation. (A) Association between tumor size (10–20 mm, 20–30 mm, 30–40 mm, and 40–50 mm) and diagnostic tumor biopsy during percutaneous RFA. Statistical analysis was performed using the Chi square test. (B) Association between visibility at ultrasonography and diagnostic tumor biopsy during percutaneous RFA. Statistical analysis was performed using the Fisher Test. (C) Association between localization based on the liver segmentation (segment I to VIII) and diagnostic tumor biopsy during percutaneous RFA. Statistical analysis was performed using the Chi square test. RFA, radiofrequency ablation.

Among the 302 nodules with an available tumor biopsy, 200 biopsies (66%) were considered diagnostic, defined by the presence of HCC, cholangiocarcinoma (CCA), hepatocholangiocarcinoma (cHCC-CCA), or dysplastic nodules at histology (Table 2). The percentage of diagnostic biopsies correlated with tumor size: 53% between 10 and 20 mm, 74% between 20 and 30 mm, 82% between 30 and 40 mm and 88% between 40 and 50 mm (*p* <0.0001; Fig. 2A). Nodules not visible on ultrasonography were associated with a lower rate of diagnostic tumor biopsies (39% *vs.* 69%, *p* = 0.003; Fig. 2B). Moreover, nodule localization influenced the likelihood of obtaining a diagnostic tumor biopsy, with fewer diagnostic biopsies in nodules located in segments I, II, VIII, and VII (50–60%) compared with other segments (67–80%, *p* = 0.049; Fig. 2C).

### Modification of diagnosis according to tumor biopsy and impact on prognosis

**Fig. 3:**
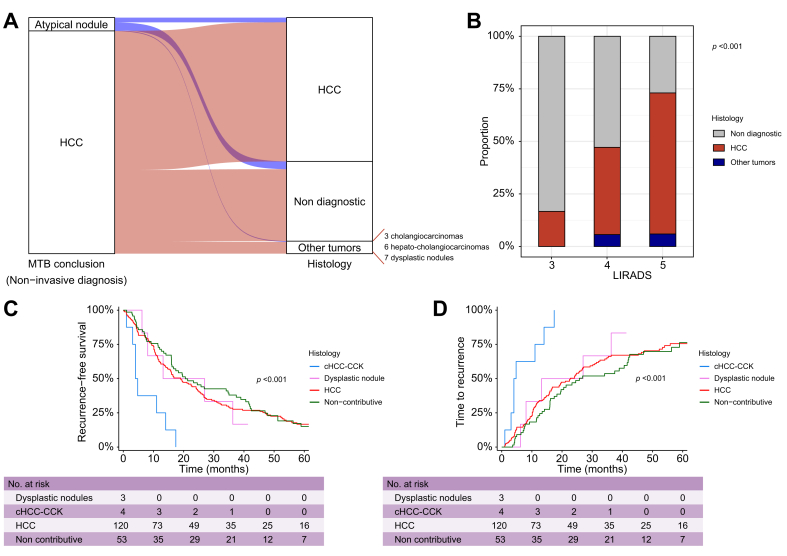
Histological diagnosis on tumor biopsy as per percutaneous radiofrequency ablation and prediction of prognosis. (A) Alluvial plots representing the concordance between the diagnosis of multidisciplinary tumor board (HCC *vs.* atypical nodules) and the results of tumor biopsy performed during percutaneous RFA. (B) Distribution in frequencies of the results of the tumor biopsy (non-diagnostic *vs.* HCC *vs.* other types of tumor) according to the LI-RADS classification (LI-RADS 3, n = 12; LI-RADS 4, n = 53; and LI-RADS 5, n = 219). Statistical analysis according to the Chi square test. (C,D) Association between histological diagnosis (HCC *vs.* dysplastic nodules *vs.* CCK and cHCC-CCK *vs.* non-diagnostic biopsy) and (C) recurrence-free survival and (D) time to overall tumor recurrence, using Kaplan–Meier method and the log rank test. The numbers at risk are represented under the x-axis. CCK, cholangiocarcinoma; cHCC-CCK, hepatocholangiocarcinoma; HCC, hepatocellular carcinoma; LI-RADS, Liver Imaging Reporting and Data System.

First, we compared the conclusion of the prior MTB regarding tumor diagnosis (285 HCC and 17 atypical nodules) with the final histological diagnosis derived from tumor biopsy performed during ablation. On histology, 102 biopsies were non-diagnostic, 184 identified HCC, three CCA, six cHCC-CCA, and seven dysplastic nodules (Table 2, Fig. 3A). Among the nine CCK and cHCC-CCA (4% of all patients included in this study), six developed on cirrhosis, one on F3 chronic HCV, one on F2 chronic HCV, and one on F1 chronic HBV based on non-tumor liver biopsy. Eight of these nine patients were classified as cirrhotic at the initial MTB. Among the 285 nodules initially considered typical HCC by the MTB, 14 were subsequently reclassified into other histological subtypes (5% including all CCA and cHCC-CCA). Of the 17 nodules considered atypical by MTB, six were classified as HCC, one as a dysplastic nodule, and 10 biopsies were non-diagnostic.

Imaging before ablation was available for reviewing by radiologists for 284 of the 302 nodules, with classification as follows: LI-RADS 3 in 12 cases (4%); LI-RADS 4 in 53 cases (19%); and LI-RADS 5 in 219 cases (77%) (Table 2). Among the 12 nodules classified as LI-RADS 3, 10 tumor biopsies were non-diagnostic and two showed an HCC. Of the 53 nodules classified as LI-RADS 4, 28 biopsies were non-diagnostic and 22 identified HCC and three dysplastic nodules. Among the 219 nodules classified as LI-RADS 5, 59 biopsies were non-diagnostic, 148 showed HCC, and 13 identified other types of tumor (three CCAs, six cHCC-CCAs, and four dysplastic nodules) (Fig. 3B). All patients found to have CCA received adjuvant treatment by capecitabine.

Next, we assessed the impact of histological diagnosis on prognosis. Tumors reclassified as CCA and cHCC-CCA (n = 9) were associated with a shorter RFS (*p* <0.001; Fig. 3C) and higher rate of overall tumor recurrence (*p* <0.001; Fig. 3D) compared with histologically confirmed HCC, dysplastic nodules, and non-diagnostic biopsies. No significant differences were observed in OS (*p* = 0.7), local tumor recurrence (*p* = 0.054), or distant recurrence (*p* = 0.15) in patients. No significant differences occurred in terms of OS (*p* = 0.7), RFS (*p* = 0.5), global tumor recurrence (*p* = 0.3), local tumor recurrence (*p* = 0.8), and distant tumor recurrence (*p* = 0.2) in patients with a histologically proven HCC *vs.* patients with a non-diagnostic biopsy.

### Histological subtypes of HCC and prediction of prognosis

**Fig. 4:**
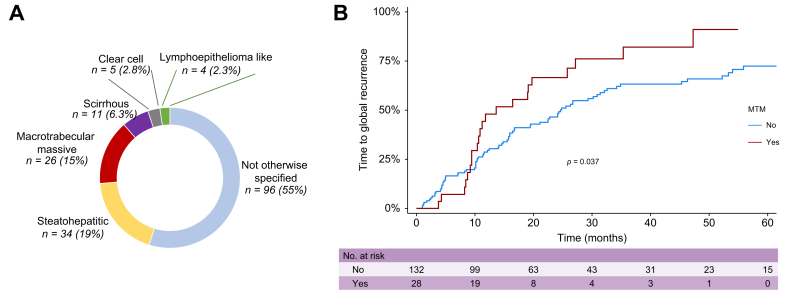
Histological subtypes of hepatocellular carcinoma and association with prognosis. (A) Distribution of the main histological subtypes (MTM, steatohepatitic, not otherwise specified, clear cell, scirrhous, and lympho-epithelioma like) of HCC (n = 176). (B) Association between MTM-HCC and time to global tumor recurrence using Kaplan–Meier method and the log rank test. The numbers at risk are represented under the x-axis. HCC, hepatocellular carcinoma; MTM, macrotrabecular massive HCC.

In total, 176 HCCs were available for pathological reviewing and were classified according to the WHO 2018 classification: 96 NOS-HCC (55%), 11 scirrhous HCC (6.3%), 34 SH-HCC (19%), four lympho-epithelioma like (2.3%), five clear cell HCC (2.8%), and 26 MTM-HCC (15%) (Fig. 4A). Moreover, HCC were classified according to OMS differentiation into well-differentiated HCC (51%), moderately differentiated HCC (33%), and poorly differentiated HCC (16%). MTM-HCC was associated with a significant higher rate of global tumor recurrence (82% at 3 years) compared with the other histological subtypes (63% at 3 years, *p* = 0.037 log rank test; Fig. 4B). We performed a competing risk analysis for tumor recurrence using death as a competitive event and the Fine and Gray method, finding that MTM-HCC was still associated with a higher risk of tumor recurrence (*p* = 0.03). By contrast, tumor differentiation was not significantly associated with tumor recurrence (*p* = 0.11). In univariate analyses, MTM-HCC (*p* = 0.039), sex (male) (*p* = 0.032), and the size of the main tumor (*p* = 0.017) were associated with higher rate of global tumor recurrence. No variables were independently associated with global recurrence in multivariate analysis (Table S2 for Cox univariate and multivariate analysis of global tumor recurrence). No significant association between MTM-HCC and OS (*p* = 0.8) and RFS (*p* = 0.09) was observed (Table S3 for Cox univariate and multivariate analysis of RFS and Table S4 for Cox univariate and multivariate analysis of OS). The other histological subtypes of HCC were not significantly associated with the different oncological outcomes (Table S2–4).

### Ability to perform molecular analysis on frozen tumor liver biopsies

Next, we assessed whether the frozen tumor samples collected during ablation could be used in the French Medicine Genomic Program to identify therapeutic targets in HCC refractory to systemic treatments using WES/RNAseq and also in the program of translational research for early HCC.

**Fig. 5:**
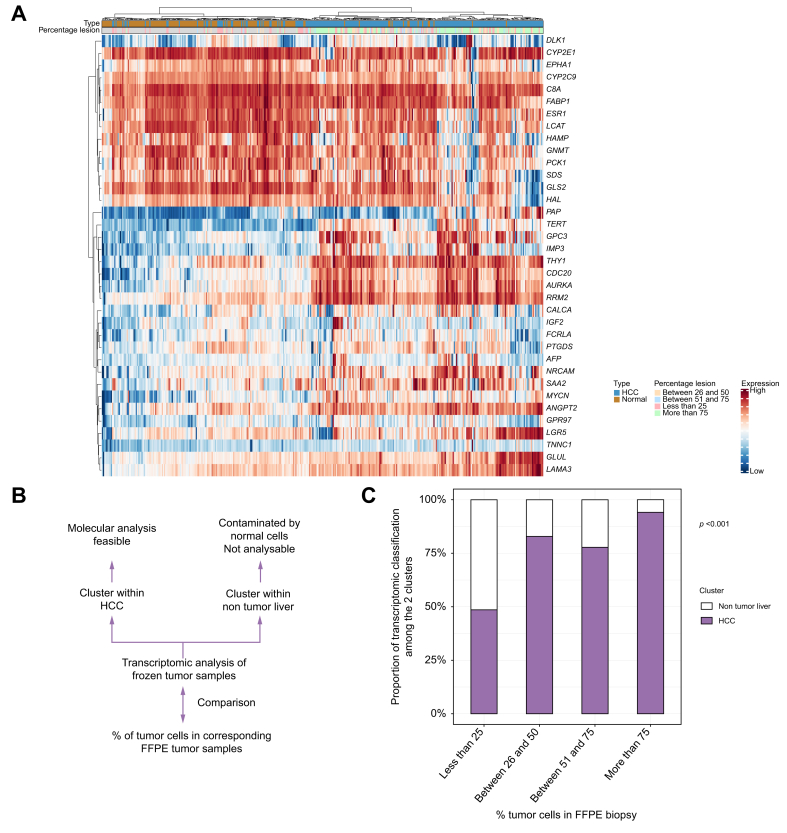
Ability to perform molecular analysis based on frozen tumor samples. (A) Heatmap of unsupervised clustering based on mRNA expression of 72 genes from frozen samples of 244 HCC collected during percutaneous ablation compared with 156 non-tumor liver. (B) Comparison of the percentages of tumor cells of the FFPE tumor samples with the results of the clustering of 244 HCC. Clustering within HCC confirmed that the frozen sample comprises tumor cells and, consequently, is analyzable by molecular biology. Clustering within non-tumor liver suggested that the frozen tumor samples is contaminated by normal cells and is not analyzable by molecular biology. (C) Distribution of the 244 frozen tumor samples of HCC among the heatmap of 244 HCC and 156 non-tumor liver samples. We divided HCC into those clustering within the tumor vs. those clustering within the non-tumor liver and compared these results with the percentage of tumor cells in the corresponding tumor on FFPE. Statistical analysis was performed using the Chi square test. FFPE, formalin-fixed paraffin-embedded; HCC, hepatocellular carcinoma.

First, among the frozen HCC samples, the quantity (median DNA level = 58 ng/ml; IQR: 21–121, and median RNA level = 97 ng/ml, IQR: 61–158) and quality of nucleic acid extracted were sufficient in 89% of cases to perform RNAseq and in 93% to perform WES. Next, we evaluated the degree of contamination of frozen tumor samples by non-tumor cells to assess the percentage of the frozen tumor samples suitable for molecular analysis. We performed unsupervised clustering based on mRNA expression of 36 genes (selected to differentiate HCC from non-tumor liver) from frozen samples of 241 HCC collected during percutaneous RFA compared with 148 non-tumor liver. We identified two main clusters: one comprising predominantly HCC samples and the other predominantly non-tumor liver samples (Fig. 5A). We then compared the percentage of tumor cells in FFPE tumor biopsies in HCC, according to the clustering results of the corresponding frozen tumor samples: samples within HCC cluster (189 HCCs with a sufficient amount of tumor cells to perform extensive molecular analysis) *vs.* samples within non-tumor liver cluster (52 HCCs contaminated by non-tumor cells, precluding tumor molecular analysis) (Fig. 5A). When <25% of tumor cells were present in FFPE, frozen tumor samples clustered within the HCC cluster in 48% of cases. By contrast, when the percentage of tumors cells was 25–75%, most frozen tumor samples clustered within the HCC cluster (78–83%). Finally, when the percentage of tumors cells was >75%, nearly all the frozen tumor samples clustered within HCC cluster (94%, *p* <0.0001; Fig. 5B).

### Assessment of the degree of fibrosis of the non-tumor liver

Finally, we compared the conclusions of the MTB regarding fibrosis of the non-tumor liver with the results of the non-tumor liver biopsies performed during percutaneous RFA. The MTB classified 220 patients (88%) as having cirrhosis and 28 (12%) as cirrhosis free. In the non-tumor liver biopsy (available in 247 patients), 18 patients were classified F0/F1 (7.3%), 13 as F2 (5.3%), 13 as F3 (5.3%, for a total of 17.9% of non-cirrhotic liver), and 203 as F4 (82%) (Table 1).

**Fig. 6:**
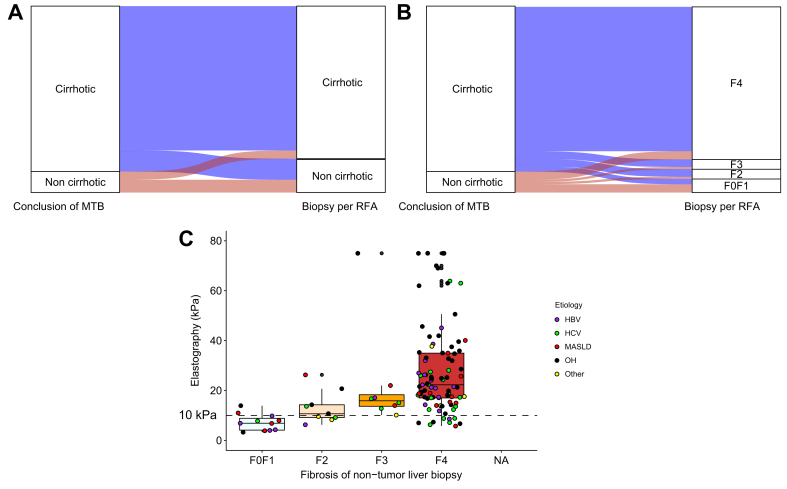
Assessment of liver fibrosis based on non-tumor liver biopsy. (A) Alluvial plots representing the concordance between the assessment of cirrhosis by the MTB and the results of tumor biopsy performed during percutaneous RFA (cirrhotic *vs.* non-cirrhotic). (B) Alluvial plots representing the concordance between the assessment of cirrhosis by the MTB and the results of tumor biopsy performed during percutaneous RFA (F0F1 *vs.* F2 *vs.* F3 *vs.* F4). (C) Association between transient elastography level, degree of fibrosis in non-tumor liver during percutaneous RFA, and the etiologies of the underlying liver disease. The Kruskall–Wallis test was used to compare the transient elastography level and degree of fibrosis of the non-tumor based on non-tumor liver biopsy. MASH, metabolic-associated steatohepatitis; MTB, multidisciplinary tumor board; OH, chronic alcohol intake; RFA, radiofrequency ablation.

A discordance between the MTB assessment and the biopsy of the non-tumor liver results was observed in 38 patients (15,3%): 27 patients initially considered to have cirrhosis were reclassified as non-cirrhotic on the non-tumor liver biopsy, while 11 patients considered non-cirrhotic were found to have cirrhosis on liver biopsy (Fig. 6A). Considering only patients classified as having cirrhosis by the MTB but later identified as F0, F1, or F2 on biopsy, we identified 17 patients with this discordance (7%) (Fig. 6B). Transient elastography values, obtained within 6 months before ablation, were available for 120 patients and correlated with the degree of fibrosis (Fig. 6C; *p* <0.0001). However, nine of the 92 patients with cirrhosis confirmed by biopsy, and with available elastography data, had a median stiffness <10 kPa (one with HBV, four with HCV, three with chronic alcohol intake, and one with metabolic syndrome, most of whom had been successfully managed medically in the preceding years).

## Discussion

This study presents the results of a prospective evaluation of tumor and non-tumor biopsies of HCC obtained during the first treatment with RFA. We propose a comprehensive, multimodal analysis that highlights the importance of biopsy in this context. This approach facilitates more precise diagnoses, better prognostic assessment, and a more detailed characterization of fibrosis in non-tumor liver tissue.

First, performing both tumor and non-tumor biopsies during percutaneous RFA was proved safe, with a 1.9% incidence of bleeding per treatment of nodules. This rate of hemorrhagic complication does not appear superior to that previously reported in a series of percutaneous ablation, suggesting the absence of additional bleeding risk related to biopsies.^20^ Only two patients required transfusion and embolization, and no deaths were recorded. Moreover, this bleeding rate aligns with the 0.5–2% incidence reported in other series of tumor biopsies of HCC outside the ablation setting.^1^ Notably, in our study, one case of hemothorax occurred in a large, 45-mm subdiaphragmatic tumor, suggesting that the bleeding resulted from the extensive RFA rather than from the biopsy itself. In addition, we observed two skin hematoma, likely more related to the ablation process than to the biopsy. There were no cases of tumor seeding in these 248 patients, with a median survival of 64.5 months, with 57% alive at 5 years. Interestingly, patients taking antiplatelet therapy did not show an increased risk of bleeding, and neither was there any correlation with thrombocytopenia, suggesting that discontinuing antiplatelet agents is unnecessary. However, these results should be interpreted cautiously because the numbers of patients in our study receiving aspirin was low.

Even among patients with platelet counts <100,000/mm^3^, no increased risk of bleeding was observed as described in other studies.^21^ This safety profile could be attributed to ablation of the biopsy tract during thermal ablations, which is likely to minimize the risk of bleeding and tumor seeding. The biopsy was performed as part of the procedure during ablation, ensuring no negative impact on treatment timing. Furthermore, because both the ablation and biopsy were conducted under general anesthesia, the patient experienced no psychological burden or pain related to the biopsy. Both safety and effectiveness of biopsies during ablation of liver tumors can also be improved because the respiratory machine provides regular breathing that could be even stopped on operator demand.

In terms of clinical applicability, most patients undergoing percutaneous ablation at our center for an initial HCC diagnosis underwent a tumor biopsy (98%). The percentage of diagnostic tumor biopsies was comparable to that reported in the literature for biopsies performed outside the ablation context, with higher performance linked to larger nodule size. Other key factors associated with successful tumor biopsies included visibility on ultrasonography and tumor location. Nodules in segments VIII and I were the most challenging to visualize, which explains the lower success rate in obtaining diagnostic biopsies from these regions. Although tumor biopsies were less effective for nodules not visible on ultrasonography, they were still diagnostic in 39% of cases, assisted by image fusion techniques to guide the procedure. This indicates that advanced radiological methods can support successful biopsies even when the target is not directly visible.^22^^,^^23^

In 5% of the tumors, tumor biopsy reclassified HCC into other histological subtypes, such as dysplastic nodules, cHCC-CCA, and CCA. Notably, nodules reclassified as CCA or cHCC-CCA were all initially classified as LI-RADS 5, underscoring that, although the specificity of LI-RADS 5 criteria for HCC diagnosis is high, it is not perfect.^2^ In a previous study,^24^ the frequency of misdiagnosis was higher, reaching 11% of LIRADS 5 nodules. However, we did not assume the diagnosis of regenerative macronodule in biopsy samples and instead based our conclusions on the non-diagnostic biopsies in our study, therefore probably reducing the frequency of misdiagnosis. Importantly, the diagnostic impact of systemic tumor and non-tumor biopsy during RFA was observed in 4% of patients, with the identification of CCA and cHCC-CCA. The reclassification of tumors as CCA or cHCC-CCA identified a small subgroup of patients with poor prognosis, emphasizing the substantial clinical importance of performing systematic tumor biopsies. Identifying CCA could also open discussions about adjuvant treatment with capecitabine, as recommended following surgery and suggested by studies on CCA treated with percutaneous ablation.^25^^,^^26^ This reclassification also has significant implications for liver transplantation, because CCA and cHCC-CCA are associated with poorer post-transplant outcomes.^27^ Furthermore, it could influence systemic treatment options for tumor recurrence.

Tumor biopsies are also valuable for identifying histological subtypes associated with distinct clinical behaviors. In our study, MTM-HCC was linked to a higher rate of tumor recurrence, as suggested by previous work.^28^ This information, derived from tumor biopsies, could be useful for stratifying the clinical management of these patients. In addition, we demonstrated that, beyond traditional FFPE samples, frozen liver tumor samples can be collected with sufficient quality and quantity of RNA and DNA in small tumors to perform WES and RNAseq in most cases. Moreover, because frozen tumor collection required a second sampling and because the percentage of tumors cells of FFPE samples could differ from the second sample collected for molecular biology, we analyzed the ability to perform molecular analysis from frozen tumor samples based on the expression of cancer genes. We found that most of the frozen tumor samples (78%) expressed cancer genes, suggesting a sufficient amount of tumor cells to perform subsequent molecular analysis. Moreover, we found that FFPE tumor samples containing <25% tumor cells were associated with clustering of most frozen tumor samples with non-tumor liver tissue, suggesting that this threshold could be used to identify samples reliable for molecular analysis. These samples could be valuable for genetic sequencing as part of the French Genomic Medicine program, enabling the personalization of systemic treatments based on genetic alterations in cases of tumor recurrence.^12^ They also hold potential for integration into translational research on early HCC.^11^

Tumor biopsies were performed alongside non-tumor liver biopsies. Having a non-tumor counterpart is useful for pathologists, allowing for more accurate interpretation of the tumor biopsy. In addition, we observed discrepancies between the presumed cirrhosis as assessed by MTB and the fibrosis degree identified through non-tumor biopsy. The degree of fibrosis was globally overestimated by the MTB compared with the results of the biopsy. Pre-ablation fibrosis assessment can be challenging in clinical practice because of factors such as the method used (*e.g.* laboratory tests, clinical evaluation, radiology, or elastography), the causes of underlying liver disease, and the impact of treatments of etiologies on fibrosis.^29^^,^^30^ In this context, non-tumor liver biopsy during ablation could provide valuable insights for a more accurate assessment of liver fibrosis. Furthermore, incorporating both tumor and non-tumor biopsies in a new type of MTB, integrating artificial intelligence, molecular biology, pathology, and advanced imaging, could improve the management of patients with HCC.^31^

However, our study has several limitations. First, it is a single-center study conducted in a tertiary center with an expert interventional radiology unit. Consequently, the results need multicentric validation in other centers managing patients with HCC. However, previous work highlighted the role of tumor biopsy in patients suspected of having HCC.^24^ Our study lacks also a cost-effectiveness analysis of tumor biopsy in clinical practice. Furthermore, we included a subset of patients with non-cirrhotic livers, for which non-invasive criteria have not yet been fully validated. Nonetheless, the use of non-invasive criteria is endorsed by the European Association for the Study of the Liver for patients with HBV without cirrhosis,^5^ and, in our study, most diagnostic modifications based on tumor biopsy occurred in patients with cirrhosis.

In conclusion, we propose a comprehensive holistic approach incorporating both tumor and non-tumor biopsies for patients with a first diagnosis of HCC undergoing percutaneous RFA. This approach offers new insights into diagnosis, prognosis, and translational research, which could be safely incorporated in the modern management of HCC using up-to-date interventional radiology approaches.

## Abbreviations

AFP, alpha-fetoprotein; BCLC, Barcelona Clinic Liver Cancer; CCA, cholangiocarcinoma; cHCC-CCA, hepatocholangiocarcinoma; CT, computerized tomography; FFPE, formalin-fixed paraffin-embedded; HCC, hepatocellular carcinoma; LI-RADS, Liver Imaging Reporting and Data System; MRI, magnetic resonance imaging; MTB, multidisciplinary tumor board; MTM, macrotrabecular-massive; NOS, not otherwise specified; OS, overall survival; R18S, ribosomal 18S; RFA, radiofrequency ablation; RFS, recurrence-free survival; RNAseq, RNA sequencing; SH, steatohepatitic; TTR, time to recurrence; WES, whole-exome sequencing; WHO, World Health Organization.

## Financial support

PN’s research is funded, in part, by the 10.13039/501100000780European Union (GENIAL, Grant agreement ID: 101096312), French 10.13039/501100001665Agence Nationale de la Recherche (France 2030 DELIVER ANR-21-RHUS-0001), and France 2030 RHU LIVER-TRACK (ANR-23-RHUS-0014). This project was supported by Association Française pour l’Étude du Foie (10.13039/501100006678AFEF) 2022 Projet Radio-moléculaire, Agence Nationale De La Recherche (ANR) 2022 SYSTHEC, Agence Nationale de Recherches sur le Sida et les Hépatites Virales (10.13039/501100003323ANRS) 2023 CSS13 HBV-LIRAGE ECTZ232901, and SIRIC CAncer Research in multiple dimensions to accelerate PrEcision Medicine (CARPEM) INCa-DGOS-Inserm-12561.

## Authors’ contributions

Contributions to conception and design: LB, MZ, OSu, J-CN. Acquisition of data: LB, NB, LDP, MZ, CC, LP, NG-C, PN, GNK, JZ-R, AD, VG, OS, OSu, J-CN. Analysis and interpretation of data: LB, MZ, CC, JZ-R, OSu, J-CN. Drafting, revising, and the manuscript content: LB, MZ, CC, OS, J-CN. Final approval of the version to be published: all the authors.

## Data availability statement

Data are available on request to the corresponding author.

## Conflict of interests

J-CN received a research grant from Bayer and Ipsen. NGC received travel and congress fees, Consulting fees or honoraria for lectures, presentations, speakers from Abbvie, Gilead, Intercept and Roche. PN has received honoraria from and/or consults for AstraZeneca, Bayer, Bristol-Myers Squibb, Eisai, Gilead, Guerbet, Ipsen, and Roche. He received research grants from AstraZeneca, AbbVie, Bristol-Myers Squibb and Eisai. None of the other authors have any conflicts of interests to declare.

Please refer to the accompanying ICMJE disclosure forms for further details.
