# Supplementary material for: Utility of tumor and non-tumor biopsies during percutaneous radiofrequency ablation for hepatocellular carcinoma
Source: JHEP Rep. 2025 Apr 22;7(9):101430. doi: 10.1016/j.jhepr.2025.101430 (PMC12355071; doi:10.1016/j.jhepr.2025.101430)
Supplement: Multimedia component 1 [file mmc1.pdf]

Utility of tumor and non-tumor biopsies during percutaneous radiofrequency ablation for hepatocellular carcinoma

Lorraine Blaise, Marianne Ziol, Claudia Campani, Nathalie Ganne-Carrie, Pierre Nahon, Gisele Nkontchou, Jessica Zucman-Rossi, Lucie Del Pozo, Nathalie Barget, Carina Boros, Elvire Desjonquieres, Alix Demory, Veronique Grando, Lorenzo Pescatori, Olivier Seror, Olivier Sutter, Jean-Charles Nault

Table of contents

Table S1……………………………………………………………………………2

Table S2……………………………………………………………………………3

Table S3……………………………………………………………………………4

Table S4……………………………………………………………………………5

Supplementary references………………………………………………………6

Table S1. Panel of 36 genes used for transcriptomic analysis by quantitative RT-PCR in order to identify HCC from non-tumor liver (taqman assay and fluidigm technology)

		Selection criteria	References
AFP	Hs00173490	Marker of malignant transformation	(1)
ANGPT2	Hs00169867	Neoangiogensis marker	(2)
AURKA	Hs01582073	Proliferation marker	(1)
C8A	Hs00175098	Proliferation marker	(1)
CALCA	Hs00266142	Proliferation marker	(1)
CDC20	Hs00415851	Proliferation marker	(1)
CYP2C9	Hs04260376	G1 G6 molecular classification	(3)
CYP2E1	Hs00559368	Hepatocyte differentiation	(4)
DLK1	Hs00171584	Marker of malignant transformation	(5)
EPHA1	Hs00178313	G1 G6 molecular classification	(3)
ESR1	Hs00174860	Hepatocyte differentiation marker	(4)
FABP1	Hs00155026	Hepatocyte differentiation marker	(4)
FCRLA	Hs00262071	Hepatocyte differentiation	(6)
GLS2	Hs00998725	Marker of malignant transformation	(7)
GLUL	Hs00374213	Wnt/B-catenin	(4)
GNMT	Hs00219089	Hepatocyte differentiation marker	(6)
GPC3	Hs00170471	Marker of malignant transformation	(8)
GPR97	Hs00416887	Proliferation marker	(1)
HAL	Hs00157887	Neoangiogenesis marker	(2)
HAMP	Hs00221783	G1 G6 molecular classification	(3)
IGF2	Hs00171254	Stem cell marker	(9)
IMP3	Hs00559907	Marker of malignant transformation	(10)
LAMA3	Hs00165042	G1 G6 molecular classification	(3)
LCAT	Hs00173415	Marker of malignant transformation	(11)
LGR5	Hs00173664	Wnt/B-catenin	(4)
MYCN	Hs00232074	Proliferation marker	(1)
NRCAM	Hs00170554	Inflammatory marker	(4)
PAP	Hs00170171	G1 G6 molecular classification	(3)
PCK1	Hs00159918	Hepatocyte differentiation	(6)
PTGDS	Hs00168748	Hepatocyte differentiation	(6)
RRM2	Hs01072069	Proliferation marker	(1)
SAA2	Hs00754237	Inflammatory marker	(4)
SDS	Hs01566038	Neoangiogenesis marker	(2)
TERT	Hs00972656	Marker of malignant transformation	(8)
THY1	Hs00174816	Stem cell marker	(12)
TNNC1	Hs00896999	Hepatocyte differentiation	(6)

Table S2: Variables associated with tumor recurrence at cox univariate and multivariate analysis

	Univariate Cox analysis			Multivariate cox analysis		
	HR	95% CI	p-value	HR	95% CI	p-value
Macrotrabecular massive HCC	1.65	1.02, 2.66	0.039	1.64	1.00, 2.68	0,048
Steatohepatitic HCC	1.30	0.83, 2.04	0.25			
Squirrheous HCC	0.61	0.27, 1.40	0.24			
Clear Cell HCC	0.82	0.20, 3.32	0.78			
Lymphoepithelioma HCC	0.60	0.15, 2.42	0.47			
Not otherwise specified HCC	0.87	0.59, 1.27	0.46			
Well differentiated HCC	ref	ref	ref			
Moderatly differentiated HCC	1.20	0.78, 1.85	0.40			
Weakly differentiated HCC	1.55	0.91, 2.62	0.11			
Gender (Male)	1.69	1.05, 2.73	0.032	0.92	0.49, 1.72	0,786
Age	1.01	0.99, 1.02	0.42			
MASLD	1.17	0.86, 1.59	0.32			
Chronic alcohol intake	1.34	0.99, 1.82	0.058	0.97	0.66, 1.43	0,885
Hepatitis B	0.81	0.53, 1.23	0.33			
Hepatitis C	0.69	0.47, 1.01	0.058	0.71	0.44, 1.13	0,149
Child Pugh B (versus A)	1.46	0.36, 5.92	0.59			
BCLC A (versus 0)	1.32	0.92, 1.89	0.13			
Creatinine (ymol/l)	1.00	0.99, 1.00	0.19			
Serum AFP (ng/ml)	1.00	1.00, 1.00	0.89			
Platelet count (/mm3)	1.00	1.00, 1.00	0.42			
Size of the main HCC	1.02	1.00, 1.03	0.017	1.01	0.99, 1.03	0,363
Non-cirrhotic liver	0.86	0.57, 1.28	0.45			

AFP= alpha foetoprotein, BCLC = Barcelona clinic liver cancer, HCC = hepatocellular carcinoma, MASLD = metabolic associated steatotic liver disease

Table S3: Variables associated with recurrence-free survival at cox univariate and multivariate analysis

	Univariate Cox analysis			Multivariate cox analysis		
	HR	95% CI	p-value	HR	95% CI	p-value
Macrotrabecular massive HCC	1.47	0.94, 2.31	0.094	1.52	0.95, 2.42	0,079
Steatohepatitic HCC	1.01	0.65, 1.57	0.95			
Squirrheous HCC	0.67	0.33, 1.38	0.28			
Clear Cell HCC	1.41	0.52, 3.82	0.50			
Lymphoepithelioma HCC	0.73	0.23, 2.31	0.60			
Not otherwise specified HCC	0.98	0.69, 1.38	0.89			
Well differentiated HCC	ref	ref	ref			
Moderatly differentiated HCC	1.24	0.85, 1.83	0.27			
Weakly differentiated HCC	1.24	0.75, 2.07	0.40			
Gender (Male)	1.48	0.98, 2.23	0.065	0.80	0.46, 1.40	0,439
Age	1.01	1.00, 1.02	0.12			
MASLD	1.31	0.99, 1.73	0.056	1.13	0.75, 1.71	0,559
Chronic alcohol intake	1.47	1.12, 1.94	0.006	1.03	0.69, 1.53	0,887
Hepatitis B	0.71	0.48, 1.06	0.093	0.92	0.49, 1.70	0,782
Hepatitis C	0.61	0.43, 0.87	0.006	0.61	0.37, 1.02	0,060
Child Pugh B (versus A)	1.84	0.59, 5.79	0.30			
BCLC A (versus 0)	1.26	0.91, 1.73	0.16			
Creatinine (ymol/l)	1.00	1.0, 1.00	0.16			
Serum AFP (ng/ml)	1.00	1.00, 1.00	0.51			
Platelet count (/mm3)	1.00	1.00, 1.00	0.80			
Size of the main HCC	1.02	1.00, 1.03	0.016	1.01	0.99, 1.03	0,295
Non cirrhotic liver	0.85	0.59, 1.23	0.40			

AFP= alpha foetoprotein, BCLC = Barcelona clinic liver cancer, HCC = hepatocellular carcinoma, MASLD = metabolic associated steatotic liver disease

Table S4: Variables associated with overall survival at cox univariate and multivariate analysis

	Univariate Cox analysis			Multivariate cox analysis		
	HR	95% CI	p-value	HR	95% CI	p-value
Macrotrabecular massive HCC	1.07	0.61, 1.88	0.82			
Steatohepatitic HCC	0.86	0.48, 1.53	0.60			
Squirrheous HCC	1.04	0.45, 2.40	0.93			
Clear Cell HCC	1.91	0.60, 6.10	0.27			
Lymphoepithelioma HCC	1.17	0.29, 4.80	0.83			
Not otherwise specified HCC	1.06	0.68, 1.65	0.81			
Well differentiated HCC	Ref	Ref	Ref			
Moderatly differentiated HCC	1.12	0.69, 1.81	0.65			
Weakly differentiated HCC	0.89	0.44, 1.78	0.74			
Gender (Male)	1.26	0.72, 2.20	0.42			
Age	1.02	1.00, 1.04	0.037	1.01	0.99, 1.03	0,171
MASLD	1.61	1.12, 2.30	0.010	1.21	0.82, 1.78	0,33
Chronic alcohol intake	1.79	1.23, 2.59	0.002	1.56	1.08, 2.28	0,019
Hepatitis B	0.71	0.42, 1.20	0.20			
Hepatitis C	0.35	0.20, 0.61	<0.001	0.42	0.23, 0.74	0,003
Child Pugh B (versus A)	1.93	0.47, 7.84	0.36			
BCLC A (versus 0)	1.06	0.70, 1.59	0.79			
Creatinine (ymol/l)	1.00	1.0, 1.00	0.59			
Serum AFP (ng/ml)	1.00	1.00, 1.00	0.20			
Platelet count (/mm3)	1.00	1.00, 1.00	0.94			
Size of the main HCC	1.01	1.0, 1.03	0.19			
Non-cirrhotic liver	0.65	0.39, 1.08	0.10			

AFP= alpha foetoprotein, BCLC = Barcelona clinic liver cancer, HCC = hepatocellular carcinoma, MASLD = metabolic associated steatotic liver disease

Supplementary references

1. Nault JC, De Reynies A, Villanueva A, et al. A hepatocellular carcinoma 5-gene score associated with survival of patients after liver resection. Gastroenterology. 2013;145:176–87.

2. Rebouissou S, Couchy G, Libbrecht L, et al. The beta-catenin pathway is activated in focal nodular hyperplasia but not in cirrhotic FNH-like nodules. Journal of hepatology. 2008;49:61–71.

3. Boyault S, Rickman DS, de Reynies A, et al. Transcriptome classification of HCC is related to gene alterations and to new therapeutic targets. Hepatology. 2007;45:42–52.

4. Bioulac-Sage P, Rebouissou S, Thomas C, et al. Hepatocellular adenoma subtype classification using molecular markers and immunohistochemistry. Hepatology. 2007;46:740–8.

5. Huang J, Zhang X, Zhang M, et al. Up-regulation of DLK1 as an imprinted gene could contribute to human hepatocellular carcinoma. Carcinogenesis. 2007;28:1094–1103.

6. Nault J-C, Couchy G, Caruso S, et al. Argininosuccinate synthase 1 and periportal gene expression in sonic hedgehog hepatocellular adenomas. Hepatology. 2018;68:964–976.

7. Chen S-S, Yu K-K, Ling Q-X, et al. The combination of three molecular markers can be a valuable predictive tool for the prognosis of hepatocellular carcinoma patients. Sci Rep. 2016;6:24582.

8. Llovet JM, Chen Y, Wurmbach E, et al. A molecular signature to discriminate dysplastic nodules from early hepatocellular carcinoma in HCV cirrhosis. Gastroenterology. 2006;131:1758–67.

9. Martinez-Quetglas I, Pinyol R, Dauch D, et al. IGF2 Is Up-regulated by Epigenetic Mechanisms in Hepatocellular Carcinomas and Is an Actionable Oncogene Product in Experimental Models. Gastroenterology. 2016;151:1192–1205.

10. Jeng Y-M, Chang C-C, Hu F-C, et al. RNA-binding protein insulin-like growth factor II mRNA-binding protein 3 expression promotes tumor invasion and predicts early recurrence and poor prognosis in hepatocellular carcinoma. Hepatology. 2008;48:1118–1127.

11. Ouyang G, Yi B, Pan G, Chen X. A robust twelve-gene signature for prognosis prediction of hepatocellular carcinoma. Cancer Cell Int. 2020;20:207.

12. Sun Y-L, Yin S-Y, Xie H-Y, et al. Stem-like cells in hepatitis B virus-associated cirrhotic livers and adjacent tissue to hepatocellular carcinomas possess the capacity of tumorigenicity. J Gastroenterol Hepatol. 2008;23:1280–1286.
